# Association of diuretics with falls and wrist fractures: a Mendelian randomization study

**DOI:** 10.3389/fpubh.2024.1381486

**Published:** 2024-10-25

**Authors:** Fei Liu, Jun-ze Dai, Xiao-xi Deng, Ren-shuang Cao, Yong-zhong Cheng, Chao-lu Wang

**Affiliations:** ^1^Department of Orthopedics, Wangjing Hospital of China Academy of Chinese Medical Sciences, Beijing, China; ^2^Department of Pneumology, Wangjing Hospital of China Academy of Chinese Medical Sciences, Beijing, China

**Keywords:** diuretics, falls, wrist fractures, Mendelian randomization, genome-wide association studies

## Abstract

**Background:**

The association between diuretics and falls in older adult has been reported in previous studies, but discrepancy remains between the different types of diuretics. The association of diuretics with the risk of wrist fractures due to diuretics is also unclear. Therefore, in this study, we determined the association of diuretics with falls and wrist fractures by Mendelian randomization.

**Methods:**

We used a two-sample Mendelian randomization (MR) approach to evaluate the effects of the loop diuretics\potassium-sparing diuretics\thiazide diuretics (LDs\PSDs\TDs) on the risk of falls and wrist fracture using the three diuretic-associated genetically-predicted single nucleotide polymorphisms (SNPs) as genetic tools. The inverse variance weighting (IVW) method was used as the main evaluation method, with odds ratio (OR) as the evaluation criterion. Additionally, weighted median (WME), MR-Egger, weighted mode (WM) and simple mode (SM) methods were used together for the MR analysis, and sensitivity analyses were performed to assess the robustness of the main results.

**Result:**

A total of 35 SNPs were included in this study as instrumental variables to replace LDs, PSDs, and TDs, which were 24, 7, and 4. Genetic substitutions for diuretics associated with increased risk of falls were LDs (OR = 1.012043, 95%CI: 1.001607–1.022588, *p* = 0.022337), PSDs (OR = 1.023794, 95%CI: 1.005605–1.042312, *p* = 0.010138). Genetically proxied TDs showed no association with falls, but the use of TDs showed a negative correlation with the incidence of wrist fracture (OR = 0.833, 95%CI: 0.767–0.905, *p* < 0.001). The Cochran *Q*-test showed no heterogeneity and MR-PRESSO method excluded data pleiotropy.

**Conclusion:**

Our findings suggest that the use of loop diuretics (LDs) or potassium-sparing diuretics (PSDs) increases the incidence of falls, but there is no causal relationship between thiazide diuretics (TDs) and falls, and TDs may actually reduce the risk of wrist fractures. Clinical use of diuretics necessitates vigilance and appropriate preventive measures to minimize fall-related events.

## 1 Introduction

Falls represent a significant global public health issue, with more than 40% of people over 65 years of age experiencing at least one fall per year (1), resulting in a variety of consequences including death, fractures and other problems, significantly impacting quality of life and imposing a substantial burden on healthcare systems worldwide (2, 3). Interest in the prevention and management of falls is growing with the increasing global population aging (4). Wrist fractures, particularly distal radius fractures (DRFs), are the most common type of osteoporotic fractures and are usually caused by straightening of the hand during a forward or backward fall (5). Several studies, including the guidelines for falls prevention and management for older adults by the World Falls Guidelines Task Force, emphasize the need to reduce the incidence of medication-induced falls in the older adult in clinical practice (2, 6), Medication-induced osteoporotic fractures have also long been a concern (7).

Although diuretics are widely used for the treatment of hypertension, heart failure, and kidney disease to reduce extracellular fluid volume and lower blood pressure, they may also pose some potential risks, such as inducing hypotension, electrolyte imbalances, or deterioration of kidney function (8, 9). Several studies have confirmed that the use of diuretics, especially loop diuretics (LDs), increases the risk of falls and fractures in patients (10, 11), but there is a lack of genetic evidence for this association. Therefore, there is a need to investigate whether there is evidence of association through a Mendelian randomization (MR) study. Another important question to answer is whether diuretics have a further effect on the occurrence of DRFs.

Mendelian randomization analysis is a method for causal inference in epidemiology that relies on genome-wide association studies (GWASs) and uses one or more genetic variants as instrumental variables (IVs), which can be independent of confounding factors since they are randomly assigned during gamete formation and are independent of environmental and lifestyle factors (12, 13). MR analysis has been widely used to investigate the association between drug factors and various diseases (14–16). We aimed to use two-sample MR analysis to determine whether genetically predicted use of diuretics is a risk factor for falls and wrist fractures, with the goal of providing evidence-based recommendations for fall prevention from the perspective of drug use.

## 2 Materials and methods

### 2.1 Study design

**Figure 1:**
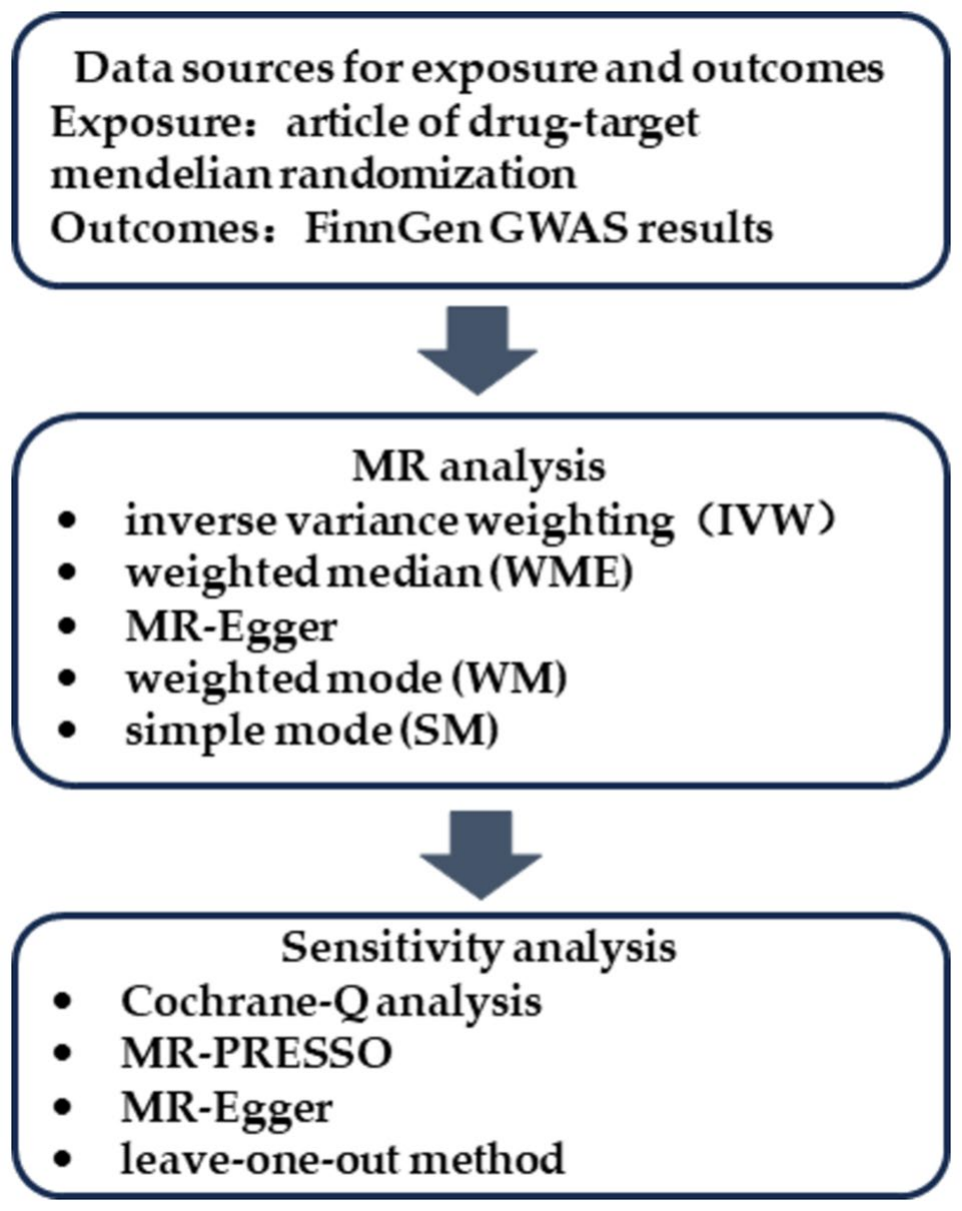
Flowchart of the MR analysis.

An overview of the study design is depicted in Figure 1. The three representative diuretics, LDs, the potassium-sparing diuretics (PSDs) and the thiazide diuretics (TDs) were chosen as exposure variables, with falls and wrist fractures as the outcome variables. The GWAS data for exposure and outcome groups were collected, and MR analysis was performed to determine the causal relationship between different diuretics and falls and wrist fractures. Three key assumptions had to be fulfilled for the MR study: (1) the IVs were strongly correlated with the exposure factors; (2) the IVs were independent of any confounders of the exposure-outcome associations; and (3) the IVs influenced the outcomes only through the exposure factors (13). All data used in this study are publicly available from the FinnGen consortium or from published articles, and do not require additional ethical approval.

### 2.2 Data sources for exposure and selection of IVs

The genetic data for the exposure variables LDs and PSDs were used in the MR analysis are from a recent study (17), which used a drug-targeted MR approach, using genetic variants near or within drug-target genes associated with systolic blood pressure (SBP) as single nucleotide polymorphisms (SNPs) for antihypertensive drugs. The study included both European and East Asian populations, and the data we used for matching outcome variables is derived solely from the European population. The study modeled diuretic in Europeans using SNPs with genome-wide significance (*p* < 5 × 10^−8^) and linkage disequilibrium (*r*^2^ < 0.1 within 100 kb) with drug target genes. For the genetic instruments for TDs, we referred to a study on the risk of kidney stones related to TDs (18) that used SLC12A3 as a predictive gene for TDs in the MR analysis. To investigate the causal relationship between diuretics and falls or wrist fractures, SNPs were used as IVs. Summary information on the data sources for the IVs used in this study is provided in Supplementary Table S1.

### 2.3 Data sources for falls and wrist fractures

Summary statistics for the associations of genetic instruments with falls and wrist fractures were obtained from the FinnGen GWAS results. The publicly available data from the R10 release (DEC 2023) includes 412,181 participants, 21,311,942 variants, and 2,408 endpoints^1^, which contains falls (102,835 cases and 309,346 controls), and fracture at wrist and hand level (12,701 cases and 366,724 controls), with all study subjects of European descent. A summary of the data sources for the outcome variables used in this study is provided in Supplementary Table S2.

### 2.4 Statistical analysis

We chose the inverse variance weighting (IVW) as the primary MR analysis (19), it is used as the main analysis method with two or more SNPs to provide relatively stable and accurate causal relationships. The weighted median (WME), MR-Egger, weighted mode (WM), simple mode (SM) methods were used together for MR analysis and to test for horizontal pleiotropy, in order to better assess the causal relationship between exposure and outcome. The WME method is consistent even when up to 50% of the information comes from invalid instrumental variables (20). The MR-Egger method is not affected by the effectiveness of IVs and reflects horizontal pleiotropy through the intercept (21). The WM method can reduce bias due to deviations in the estimation results of certain genetic variations (22). MR-PRESSO tests for horizontal pleiotropy by identifying and removing outlier IVs (23). Subsequently, the Cochrane-Q analysis was used to detect heterogeneity (24) and the leave-one-out method was used to test the MR estimation results after removing each individual SNPs. The R software (4.3.1) was used in this study, including the R packages TwoSampleMR, MR-PRESSO, and forestploter, all of which are freely available on the R software official website^2^. All reported *p*-values are two-tailed, and *p* < 0.05 is considered to indicate a significant difference.

## 3 Results

In the MR analysis results, the exposure was considered a risk factor for the outcome if OR > 1, and was considered a protective factor if OR < 1. Results are presented as odds ratios (OR) and 95% confidence intervals (95% CIs). The corresponding *p*-values were used to determine their statistical significance.

### 3.1 Association between genetically predicted diuretics and falls

**Figure 2:**
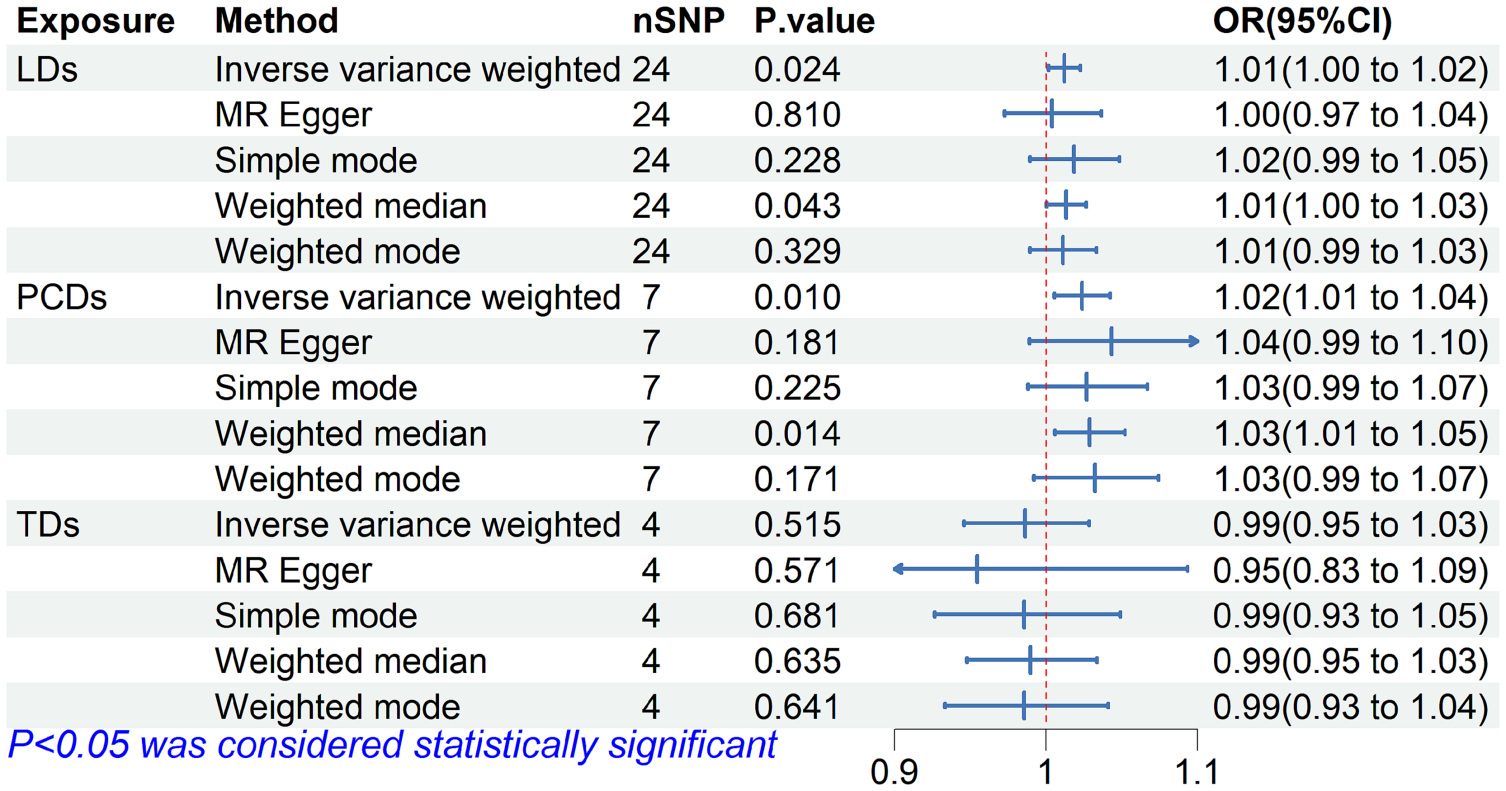
Forest plot of Mendelian randomization for the relationship between diuretics and falls. The random effects inverse-variance weighted (IVW) method was utilized in the main MR analysis.

The MR analysis results for the three influences on falls are shown in the forest plot in Figure 2, with the IVW results as the main outcome. The genetically predicted LDs (*p* = 0.024) and PSDs (*p* = 0.010) are associated with an increased risk of falls, while the results did not reveal a significant effect of TD on falls. The leave-one-out analysis also indicated that the basic causal effect did not change regardless of which SNP was left out, confirming the robustness of the MR analysis (Supplementary Figure S1). Our analysis did not find significant heterogeneity as indicated by the Cochrane-Q statistic, and there was no significant evidence of pleiotropy in the MR-Egger regression, as indicated by the MR-Egger intercept test. Additionally, the MR-PRESSO analysis results suggested no horizontal pleiotropy in the data (Supplementary Table S3).

### 3.2 Association between genetically predicted diuretics and wrist fractures

**Figure 3:**
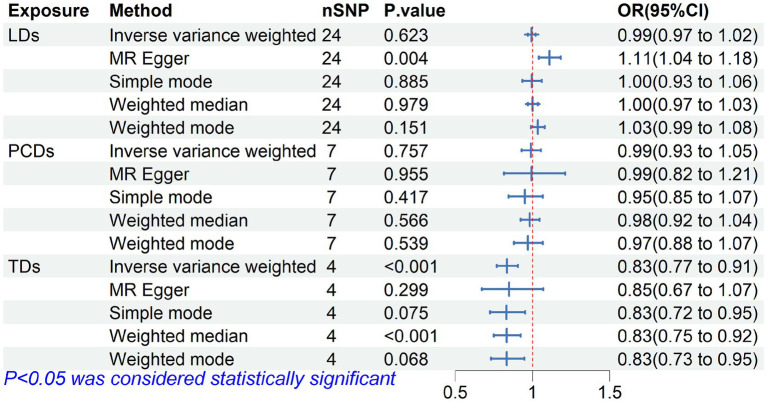
Forest plot of Mendelian randomization for the relationship between diuretics and wrist fractures. The random effects inverse-variance weighted (IVW) method was utilized in the main MR analysis.

We further analyzed the causal relationship between genetic instruments for diuretics and wrist fractures. The preliminary results suggest that the use of TDs can on average reduce the risk of wrist fractures by 16.67%, while the use of LDs and PSDs IVs are not associated with wrist fractures, as shown in Figure 3. The statistical results are presented in Supplementary Table S4 and Supplementary Figure S2.

## 4 Discussion

This study revealed the causal relationships between three types of genetically proxied diuretics and the risk of falls and wrist fractures. MR analysis predicted that the use of LDs and PSDs increases the risk of falls, and neither of the two diuretics has an impact on the risk of wrist fractures. However, these findings mainly indicate a potential association. The use of TDs does not affect the incidence of falls, but it can reduce the risk of wrist fracture by an average of 16.67%.

Diuretics are considered as clinically important antihypertensive drugs, and the latest guidelines emphasize their significance in the treatment of antihypertension, particularly in refractory hypertension where they play an irreplaceable role (25). The three most commonly used diuretics have different mechanisms of action, and irrational use will cause some complications. Falls have become the most unpredictable injuries in the older adult and are closely associated with psychiatric disorders, cerebrovascular disease, and skeletal muscle disease, etc. Several meta-analyses studies have demonstrated the risk of falls from neurological medications, cardiovascular/circulatory system medications, and other medications (10, 26–28). Among them, LDs have been identified as a significant factor in increasing the risk of falls, which is consistent with our study. First, it is evident that the use of diuretics can easily lead to insufficient blood volume, particularly in older adult patients, resulting in orthostatic hypotension or syncope, thereby increasing the risk of falls (29). Second, the action of all diuretics affects the electrolyte balance. LDs such as furosemide inhibit the active reabsorption of NaCl by blocking the Na + -K + -Cl− co-transporter, resulting in hyponatremia, hypokalemia, and hypocalcemia (30), causing muscle pain, spasms, and instability in walking, leading to falls. Additionally, PSDs may cause hyperkalemia, and TDs increase calcium reabsorption, leading to hypercalcemia. Bumetanide (an LD) impairs myogenic differentiation and exercise-induced muscle hypertrophy, leading to muscle wasting (31), and also has a sedative effect (32), which can cause patients to faint after taking the medication, thus increasing the risk of falls. Finally, the frequency of patient urination increases after treatment with diuretics, studies have shown that environmental factors such as bed height, toilet handles, and slippery and uneven surfaces, also influence the probability of falling (3), leading to more falls during the process of walking from the bed to the toilet. Although we concluded that diuretics increased fracture risk, the OR value was modest, compared to 1.185 for diuretics (33) or 1.36 for LDs (10). In our study, TDs had no impact on the risk of falls, which remains controversial. A study by Ravioli et al. (34) suggests that the risk of falls increased after the use of TDs, while another meta-analysis combining multiple studies found no evidence of such risk (10). World guidelines for falls prevention and management for older adults recommend to identify and appropriate deprescribing fall risk increasing drugs (6).

In orthopedic research, the risk of wrist fractures, represented by DRFs, is associated with falls (5). Although diuretics increase the risk of falls, current research indicates that they do not increase the incidence of DRFs (35), but the existence of a positive correlation between TDs and hip fractures has been reported, and the effect disappears within 4 months after use is discontinued (36). In contrast, our MR analysis suggests that the use of TDs can reduce the occurrence of wrist fractures, possibly due to their positive effect on bone health and structure. The direct effects of TDs include stimulating the production of runt-related transcription factor 2 (RUNX2) and osteopontin to promote osteoblast differentiation while reducing the production of osteoclasts (37). Indirectly, TDs increases blood calcium (38) and enhances the formation of mineralized nodules (39). In certain patient populations, such as those with hyperparathyroidism and hypercalciuria, TDs can normalize parathyroid hormone levels, thereby slowing down bone resorption and the dissolution of bone matrix (40). DRF is a common osteoporotic fracture, and prevention of osteoporosis is an effective measure to reduce this type of fractures. An MR study found a positive correlation between TDs and calcaneal bone density (41), which partly explains the reduction in the occurrence of wrist fractures with TDs.

Fall prevention has become a critical public health issue, with peak of concentrations reached within 0.5–2 h of taking LDs (42). The daily dose of diuretics needs to be strictly controlled, as it is significantly associated with adverse outcomes (43). The side effects of the drug must be closely monitored, such as hypokalemia, upright hypotension and dizziness, must be closely monitored, with the moment of standing up being the most dangerous. Additionally, regular review of electrolyte levels for medication adjustment is an effective measure to prevent falls (30). Regarding the issue of increased urination frequency, the authors believe that emptying the bladder before taking the medication can help avoid an increased risk of falling after taking the medication. If the patients feel weakness in the limbs after taking the medication, it is best to use a bedside commode. For patients at high risk of hypertension combined with osteoporosis, TDs are more recommended in the selection of diuretics (44).

This study has inherent limitations in MR analysis. For instance, the genetic instrumental variables obtained from GWAS may not be the same as those in clinical practice, thus the main purpose of our MR analysis was to assess causality rather than quantify the effect size, the influence of the study’s objective on clinical practice still needs to be further demonstrated. Although evidence of the association of the use of diuretics being with falls was observed in our study, it was better suitable for older persons, the estimation of the genetic effects of the medication cannot be explained as the clinical effects of the medication, especially in the case of initial use or dosage adjustments. The MR analysis cannot evaluate the effect indicators of the risk of falls for the choice between intravenous injection or oral medication, or for combination therapy with multiple drugs. For non-European populations, we did not perform the analysis due to the lack of appropriate outcome data, and the effects of this result on other ethnic groups remain to be investigated.

## 5 Conclusion

The results of our study indicate that diuretics, a classical medication, such as LDs and PSDs increase the risk of falls occurrence but does not have an effect on wrist fractures incidence. In contrast, TDs may not be causally related to the occurrence of falls and may reduce the risk of wrist fractures. MR analysis is not limited by the short follow-up period of clinical trials. It is necessary to further investigate the impact of diuretics on falls and guide clinical practice to avoid diuretic-induced falls as much as possible through rational use of drugs, patient education, and other means.

## Data Availability

The datasets presented in this study can be found in online repositories. The names of the repository/repositories and accession number(s) can be found in the article/Supplementary material.

## Footnotes

^1^https://r10.finngen.fi/, accessed on 07 January 2024.

^2^https://www.r-project.org/
