# Supplementary material for: Association of diuretics with falls and wrist fractures: a Mendelian randomization study
Source: Front Public Health. 2024 Oct 25;12:1381486. doi: 10.3389/fpubh.2024.1381486 (PMC11543579; doi:10.3389/fpubh.2024.1381486)
Supplement: Supplementary file 1 [file Data_Sheet_1.docx]

Supplementary Material

## Supplementary Figures

**
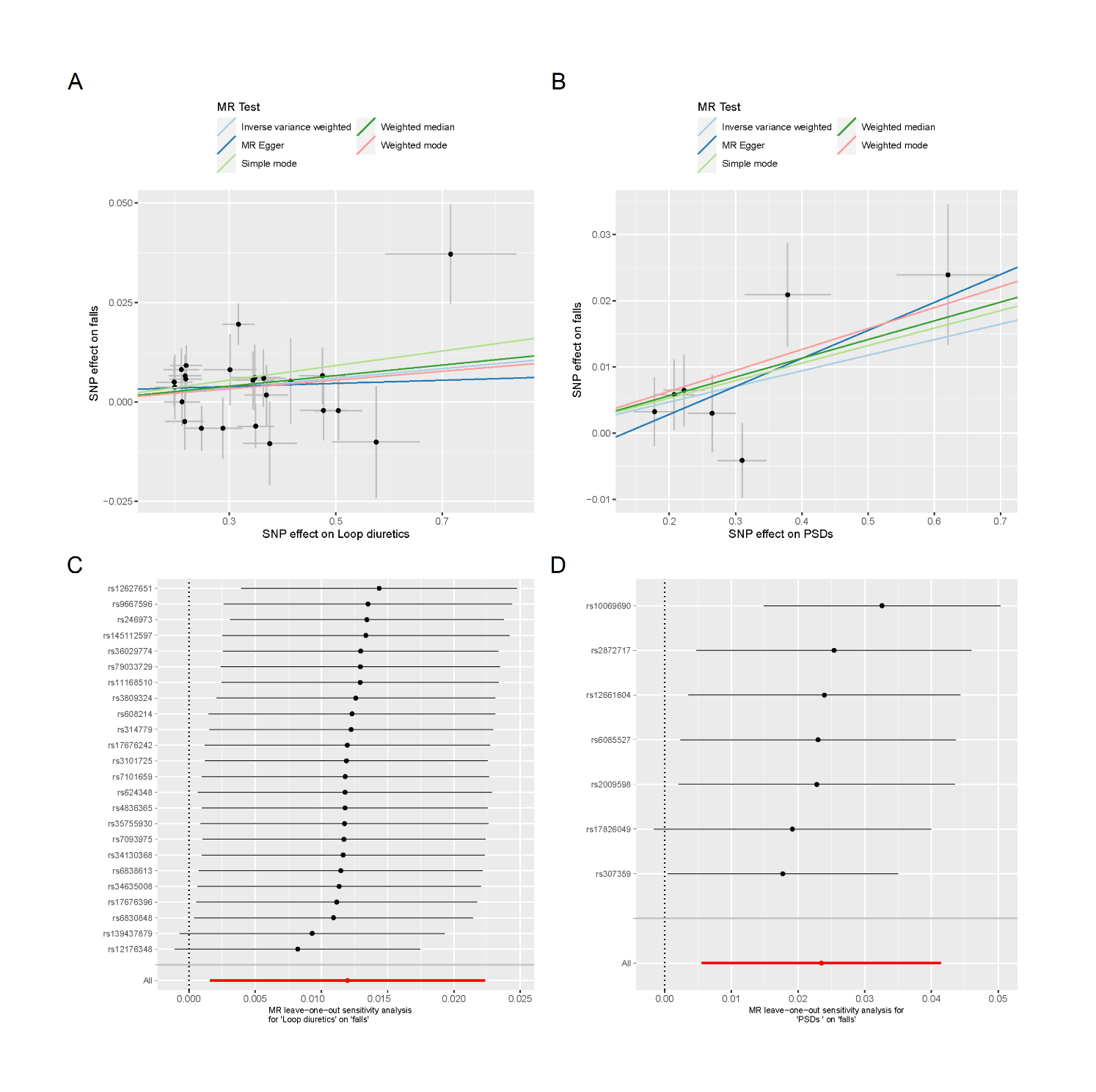
**

**Supplementary Figure 1.** Scatter plots (A, B) and leave-one-out result (C, D) depicting the results of the association between association between LDs/PSDs and falls


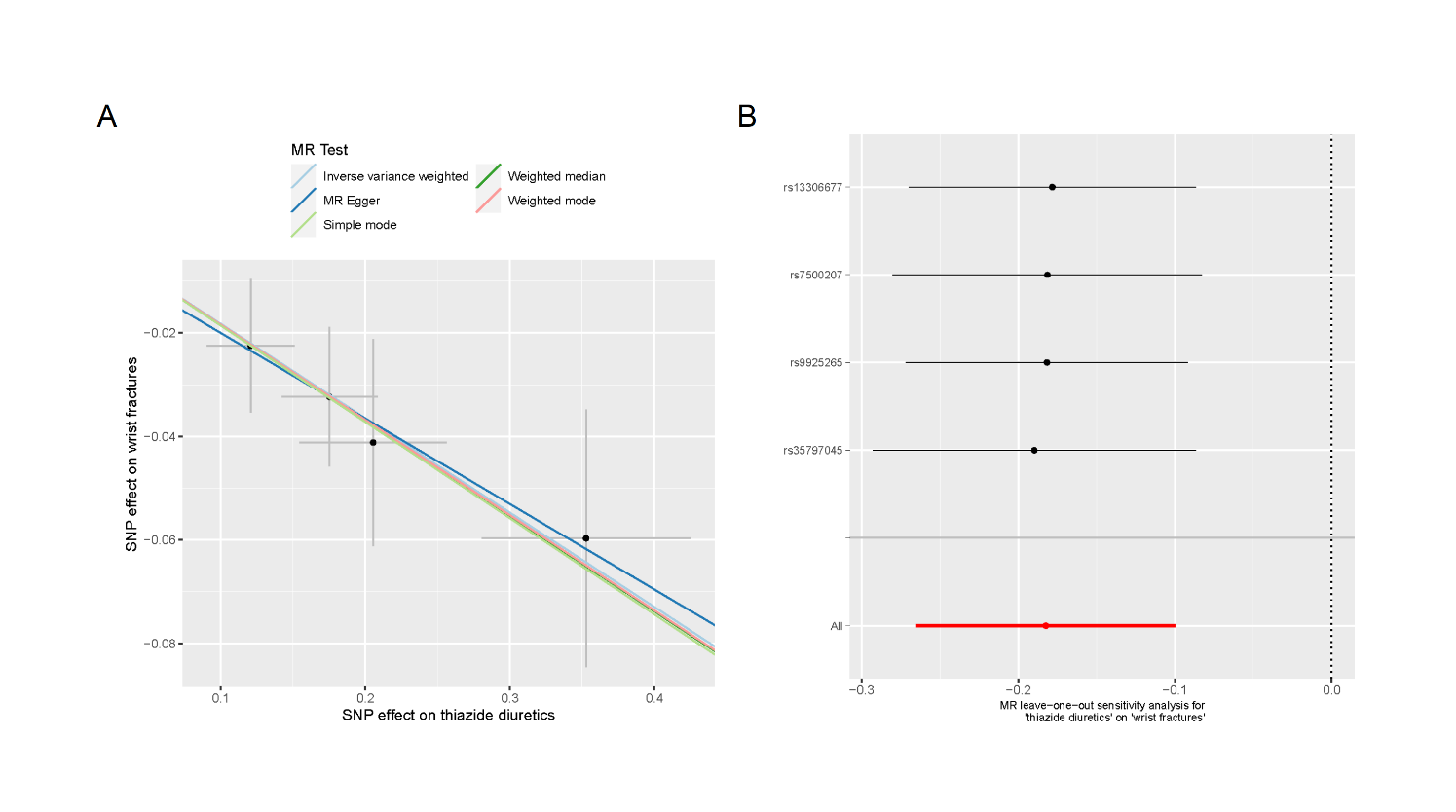


**Supplementary Figure 2.** Scatter plots (A) and leave-one-out result (B) depicting the results of the association between association between TDs and wrist fractures
